# Recent Developments on Using Nanomaterials to Combat *Candida albicans*

**DOI:** 10.3389/fchem.2021.813973

**Published:** 2021-12-23

**Authors:** Bingxin Li, Luyao Pan, Haofeng Zhang, Lingping Xie, Xi Wang, Jiahui Shou, Yu Qi, Xiaojian Yan

**Affiliations:** ^1^ Department of Gynecology, The First Affiliated Hospital of Wenzhou Medical University, Wenzhou, China; ^2^ Wenzhou Institute, University of Chinese Academy of Sciences, Wenzhou, China

**Keywords:** vaginal candidiasis, *Candida* albicans, nanomaterials, antifungal applications, biofilms

## Abstract

Vaginal candidiasis (VC) is a common disease of women and the main pathogen is *Candida albicans* (*C. albicans*). *C. albicans* infection incidence especially its drug resistance have become a global health threat due to the existence of *C. albicans* biofilms and the low bioavailability of traditional antifungal drugs. In recent years, nanomaterials have made great progresses in the field of antifungal applications. Some researchers have treated fungal infections with inorganic nanoparticles, represented by silver nanoparticles (AgNPs) with antifungal properties. Liposomes, polymeric nanoparticles, metal-organic frameworks (MOFs), and covalent organic frameworks (COFs) were also used to improve the bioavailability of antifungal drugs. Herein, we briefly introduced the recent developments on using above nanomaterials to combat *C. albicans* in antifungal applications.

## Introduction

Vaginal candidiasis (VC) is the second common mucosal infection of the vagina, with around 80–95% cases caused by *Candida* species, especially *Candida albicans* (*C. albicans*). About 75% of women occur at least once in their lifetime, and at least twice in more than half of patients. Around 5–8% of patients will develop recurrent vulvovaginal candidiasis (at least 4 times per year) (Anderson et al., 2004; Sobel, 2007). 80–95% of these pathogens are *C. albicans*.

**FIGURE 1:**
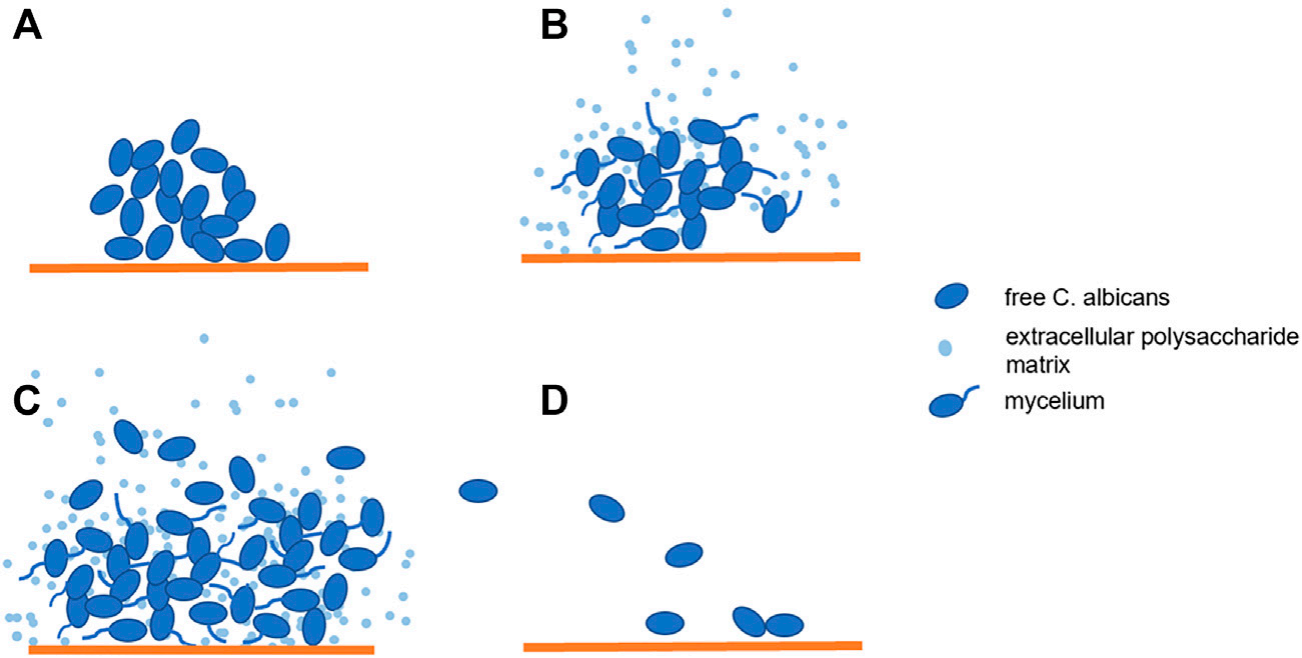
Biofilm Formation: **(A)**. *Candida albicans* adhere to the substrate; **(B,C)**. The cells proliferated and formed mycelia while extracellular polysaccharide matrix accumulated; **(D)**. The beginning of new biofilm formation.

*C. albicans* is an opportunistic pathogen that colonizes the mucous membranes of the skin and gastrointestinal tract in 30–50% of normal healthy individuals. *C. albicans* only becomes pathogenic when the local or systemic defense mechanism is damaged. The most common infection sites are oropharynx, esophagus and vagina (Johal et al., 2016). *C. albicans* has three forms: spore, pseudohyphae and hyphae (Molero et al., 1998). The morphological transformation from spore to mycelium also means that *C. albicans* has transformed from symbiotic state to pathogenic state, while pseudohyphae is the transitional form of both (Talapko et al., 2021). The hypha invades host tissues through active penetration and induces endocytosis. In this process, hypha synthesizes and secretes various proteins and form biofilms. Actually, most *C. albicans* infections are associated with the formation of biofilms on the host or abiotic surfaces (Maza et al., 2017). Biofilm is a highly structured and complex microbial community whose formation consists of several successive stages (Figure 1). In the first stage, free *C. albicans* adhere to the substrate, forming the basal layer of the biofilm. Then albicans cells form elongated projections that continue to grow into filamentous hyphal form. This process is known as the cell proliferation and filamentation, and is also the sign of the beginning of biofilms forming. Extracellular polysaccharide matrix accumulates during the mature stage. Finally, unattached cells disperse between tissues and begin the formation of new biofilms (Talapko et al., 2021). The three-dimensional communities of these surface-attached and tightly packed fungi complicate treatment and can lead to drug resistance and persistent fungal infections (Li et al., 2018). Biofilms can achieve immune escape of fungal cells in the host (Nett, 2014; Vera-González and Shukla, 2020) and secrete a dense extracellular polymer, which can serve as a physical barrier during antifungal therapy, making most antifungal drugs difficult to pass through this barrier and less effective at their target position (Singh et al., 2018; Vera-González and Shukla, 2020; Talapko et al., 2021). In addition, studies have reported that drug resistance may also be related to the thickness of the bacterial cell wall. It has been observed that the thickness of the biofilm is twice that of free cells (Nett et al., 2007; Lima et al., 2019).

VC treatments based on standard antifungal agents such as azole, polyene and echinocandin include oral or vaginal topical administration. Transvaginal agents are absorbed through three main pathways: transcellular absorption, paracellular absorption and active transport. Transcellular and paracellular absorption rely on a intra-/extra-cellular concentration-dependent gradient and tight connections between cells; while active transport depends on vesicles or receptors on the surfaces of cells. The absorption efficiency of drugs is affected by many factors, such as the thickness of the vaginal epithelium, the viscosity of vaginal secretions, pH value and the solubility of drugs (Johal et al., 2016). In addition, drug stability, bioavailability and compliance of patients are also essentisal factors of antifungal therapy. While oral drugs are often difficult to transport to target position because of their poor solubility and bioavailability, resulting in an increased incidence of adverse reactions due to lack of drug selectivity. The common adverse reactions include gastrointestinal symptoms, nephrotoxicity and hepatotoxicity (Paul and Sharma, 2010). And oral administration often fails to produce satisfactory results for severe fungal infections (Johal et al., 2016).

**FIGURE 2:**
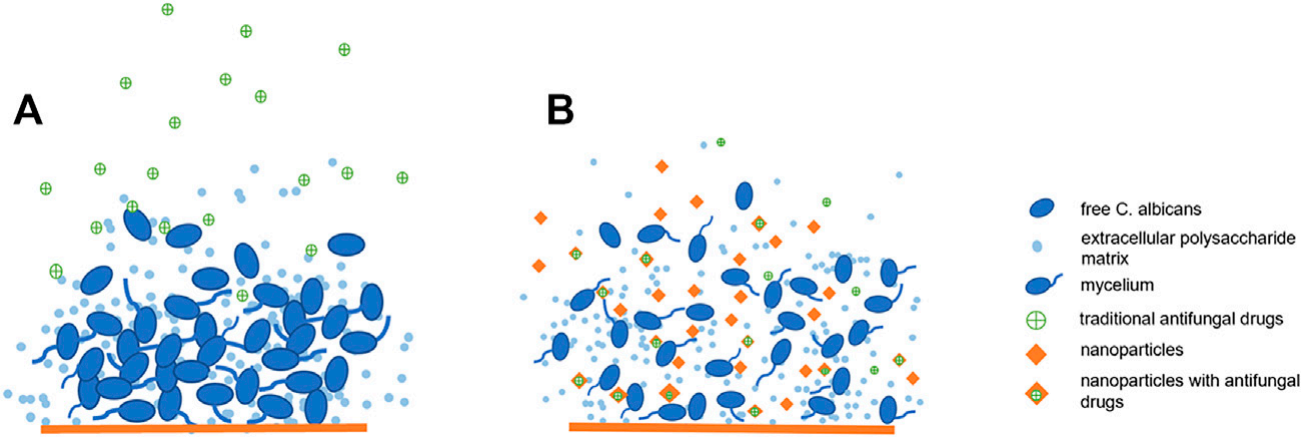
Nanoparticles have anti-biofilm effect: **(A)**. Traditional antifungal drugs have difficulty passing through biofilms: **(B)**. Nanoparticles have anti-biofilm effect and can improve the bioavailability of antifungal drugs.

Nanomaterials have developed rapidly in recent years. The application of nanomaterials in the medical field is also popular direction. Nanoparticles have the potential to carry, stabilize and protect therapeutic payloads, penetrate extracellular polymeric substances (EPS), target fungal cells, making nanoparticle therapeutics for the treatment of *candida* biofilms become a promising strategy (Figure 2). Here we will introduce some advanced developments in antifungal therapy by nanomaterials.

## State-of-the-Art Nanoparticle-Based Strategies Used in Eradicating *Candida albicans*

### Inorganic Nanoparticles

Many inorganic substances have been known to have antibacterial effects. However, due to the lack of targeting and difficult to control the release of inorganic substances, they accumulate in normal tissues and produce toxic and side effects. Inorganic nanoparticles not only have antibacterial effects, but also have the advantages of non-toxicity, hydrophilic, biocompatibility and high stability, which have been gradually paid attention to in the fields of medical imaging diagnosis and drug delivery (Spadari et al., 2017).

Silver nanoparticles (AgNPs), the diameter of which is less than 100 nm, with higher antibacterial activity and larger relative surface areas, show antibacterial activity against the target microbial biofilm at low concentrations (Monteiro et al., 2011; Takamiya et al., 2016). AgNPs attack the membranes of yeast cells and disturb the membrane lipid bilayer, forming pores that lead to leakage of ions and other substances, dissipating the membrane potential (Kim et al., 2009). Hwang et al. found AgNPs kill fungal cells by inducing the accumulation of reactive oxygen radicals, especially hydroxyl radicals (Hwang et al., 2012). Lara used transmission electron microscopy to find that the fungal cell wall was infiltrated and the outer cell wall of the structural layer was destroyed after the treatment of silver nanoparticles. This indicates that the anti-biofilm effect of AgNPs is achieved via cell wall disruption (Lara et al., 2015). In addition, AgNPs can also accumulate outside fungal cells and release silver ions through the interaction between cell components and ionic silver, inducing cell death (Vazquez-Muñoz et al., 2014). Takamiya et al. combined AgNPs with acrylic resin (a material with strong bending strength and antifungal effect) and found that the effect of antifungal biofilm was increased, while the bending strength of acrylic resin itself as usual. In addition, 60 days after the recombinant material was implanted into the subcutaneous tissue of mice, there was no significant difference in inflammatory response compared with control group, suggesting that the AgNPs were biocompatible (Takamiya et al., 2021).

Bismuth is another metallic element, whose compounds have been used in medicine for more than two centuries (Vazquez-Munoz et al., 2020a). Recently several bismuth compounds have been found to have antibacterial and antifungal activities (Ferraz et al., 2013). Some reports indicated that bismuth nanoparticles (BiNPs) display promising anti-microbial activity on bacteria, fungi, and protozoan. However there are few articles about antibacterial activity of nano-bismuth due to their difficulties in synthesizing and low stability. Most recently an American group has described a method for the fast, facile, and inexpensive synthesis of PVP-coated bismuth nanoparticles (PVP-BiNPs), which can be used even in a non-specialist laboratory (Vazquez-Munoz et al., 2020b). PVP-BiNPs are more stable than the bismuth (III) ions and the bismuth-BAL complexes. PVP-BiNPs have been proved to have strong antibacterial activity against both pathogenic *Staphylococcus aureus* (*S. aureus*) and conditionally pathogenic yeast *C. albicans*, whether under plankton or biofilm growth conditions (Vazquez-Munoz et al., 2020a). However, the antibacterial mechanism of BiNPs is unclear and remains to be further studied.

Zinc oxide nanoparticles (ZnO-NPs) have been widely used in many fields due to their optical transparency, electrical conductivity, piezoelectric properties, non-toxicity, wide applicability, low cost and good stability. What’s more, they can also be used to fight bacteria and fungi. ZnO-NPs can be successfully synthesized by homogeneous precipitation method. ZnO-NPs with small particle size and large specific surface area had strong antifungal activity. The antibacterial activity of ZnO-NPs against *C. albicans* was detected by disc diffusion susceptibility method. It was observed that the inhibition band increased with the increase of ZnO-NPs concentration (Sharma and Ghose, 2015).

Inorganic nanoparticles have achieved satisfactory results in antifungal treatment. However, their high requirements for preparation conditions, high production costs, poor stability and other shortcomings hinder the process of large-scale clinical use. In addition, the antibacterial mechanism of inorganic nanoparticles remains to be further studied.

### Liposomes

Liposomes (Garg and K. Goyal, 2014) are mainly composed of non-ionic lipid molecules such as phospholipids and cholesterol, which could form thin lipid films or lipid cakes. In the process of agitation, hydration of lipid will unlock and self-close, forming large multilamellar vesicles. The existence of lipid bilayer and aqueous medium make liposomes both lipophilic and hydrophilic, which means they can be used to encapsulate and deliver both hydrophilic and lipophilic drug molecules. What’s more, liposomes have good solubility and stable structure, which can reduce the phagocytosis of macrophages on drugs, maintain blood drug concentration, reduce the influence of microenvironment on drugs, promote the intracellular transmission of drugs (Garg and K. Goyal, 2014). Encapsulation of antifungals into liposome nanoparticles, which may improve the transport and action efficiency of antifungal drugs, has become one of the research hotspots in recent years.

Liposomal amphotericin B is the first commercially formulated nanoparticle system for antifungal drugs. Host toxicity of amphotericin B was significantly reduced in liposomal amphotericin B (Olson et al., 2008). The ultra-deformable amphotericin B liposomes containing surfactants have stronger epidermal permeability (Perez et al., 2016). VERA-GONZÁLEZET et al. successfully encapsulated anidulafungin (an echinocandin antifungal drug) into liposomes. These liposome formulations can inhibit planktonic *C. albicans* growth with a similar minimum inhibitory concentration (MIC) to anidulafungin, but localize to the *candida* cells more quickly and perform better in penetrating fungal biofilms compared with free anidulafungin. What’s more, in comparison with free agents, using liposomes at the same concentration 2 days before infection significantly improved survival rate (Vera-González et al., 2020).

Actually, liposomes can simultaneously transport two or more drugs to the targeted position. For example, C. Carbone et al. have created a liposome formulation which combined with solid lipid nanoparticles, clotrimazole (CLZ) and alphalipolic acid (ALA), marked as CLZ-ALA-loaded SLN. Mean size of nanoparticles were below 150 nm and exhibited slight negative or highly positive zeta potential values, so that the nanoparticles can remain stable in the presence of cationic resin. Furthermore, they confirmed the formation of SLN was able to release drug slowly but steadily *in vitro*. This research indicates that the dual-delivery SLN developed with ALA protective antifungal agents may be a promising strategy for the treatment of *candida* (Carbone et al., 2020).

Despite the promising prospect of liposome nanotechnology, its low solubility, short half life, poor stability, allergic reaction, high production cost and low production efficiency are still problems that need to be solved urgently (Garg and K. Goyal, 2014).

### Polymeric Nanoparticles

Polymeric nanoparticles (polymeric NPs) are composed of organic polymers. According to their structural forms, they can be roughly classified as nanocapsules and nanospheres. Nanocapsules consist of a core that dissolves the drug and a polymer shell that controls its release. Nanospheres are a continuous network of polymers in which drugs are stored inside or adsorbed to the surface (Zielinska et al., 2020). Chitosan and poly (lactic-*co*-glycolic acid) (PLGA) are two common materials used to prepare polymer nanoparticles.

Chitosan is a kind of biocompatible, biodegradable, selective non-toxic cationic polysaccharide (Ing et al., 2012; Wimardani et al., 2012), which has certain antifungal activity (Dhillon et al., 2014). These advantages make it a popular material the preparation of nanoparticles in recent years. Ferulic acid (FA) and its derivatives have been found to have antibiofilm potential against *C. albicans*. However, the applicability of FA is limited by its low permeability and instability. Additionally, FA is a plant phenolic compound, which is unable to distinguish between diseased and healthy cells, and may lead to undesirable side effects. Coating them with nanoparticles in polymers allow them to accumulate at the specific targets, preventing harmful effects on normal tissues (Park et al., 2010). Panwar et al. composed FA encapsulated chitosan nanoparticles (FA-CSNPs) exhibited the positive zeta potential, which allowed them to bind to negatively charged fungal membranes and thus destroed their membrane integrity, resulting in intracellular material leakage and biofilm inhibition (Panwar et al., 2016). Chitosan also binded with trace elements to inhibit the normal growth of fungi (Roller and Covill, 1999). In addition, some scholars proposed that chitosan can penetrate fungal cell wall and bind to its DNA, thus inhibiting the synthesis of mRNA and the production of essential fungal proteins and enzymes (Kong et al., 2010).

Curcumin is one of the main components extracted from turmeric, a perennial flowering plant in the rhizome of Zingiberaceae. Recently, curcumin loaded chitosan nanoparticles (CSNP-CUR) have been synthesized and used as antibacterial agents (Samrot et al., 2018). CSNP-CUR has been confirmed to have stronger antimicrobial activity and biofilm resistance than free curcumin. Furthermore, Ma et al. found that CSNP-CUR also showed excellent antibacterial, antifungal and antibiofilm effects on the polymicrobial biofilm, indicating that CSNP-CUR may be a novel strategy for polymicrobial biofilm related infections (Ma et al., 2020).

In addition, other chitosan based nanoparticles with antifungal activity and biofilm potential have been reported, such as chitosan-pentasodium tripolyphosphte (CS-TPP) nanoparticles (Ing et al., 2012) and chitosan based zinc oxide nanoparticles (Dhillon et al., 2014).

Poly (lactic-*co*-glycolic acid) (PLGA) is another popular material for the preparation of polymeric nanoparticles. Different from chitosan, PLGA is a synthetic polymer organic material, and its advantages are similar to those of chitosan (Cartiera et al., 2009).

Ketoconazole is a common antifungal drug in clinical practice. However, due to its low bioavailability, conventional doses of ketoconazole are difficult to achieve satisfactory antifungal activity, while large doses are likely to cause severe allergic and skin reactions. Sadozai et al. prepared ketoconazole-loaded PLGA nanoparticles (Keto-PLGA NPs) by emulsion/solvent evaporation method. This formulation succeeded in enhancing the solubility of drug. Under experimental conditions, they found that the sustained release of ketoconazole from the nanoparticles resulted in a weaker fungal inhibition than the same dose of free ketoconazole. However, when Keto-PLGA NPs interacted with AgNPs, the largest growth inhibition region is produced, indicating a strong synergistic effect between two materials (Sadozai et al., 2020), which is consistent with previous reports of synergistic effects between AgNPs and other azole-based antimycotic drugs (Sun et al., 2016).

Currently, some polymeric NPs have been successfully prepared, but current research tends to focus on their physical and chemical properties. We still lack data on their ecotoxicology and how the drug relates to the polymeric NPs.

### Metal-Organic Frameworks (MOFs) and Covalent-Organic Frameworks (COFs)

Metal-organic frameworks (MOFs) is a kind of organic-inorganic hybrid crystalline materials, containing positively charged metal ions and organic linking molecules arranged in order. MOFs structures have many good properties, such as ultra-high surface areas, porosities, biocompatibility, biodegradability and stability in water and biological media, which make MOFs a new hotspot in the field of nanocomposites (MOFs (Metal-Organic Frameworks, 2021)). Different from MOFs, COFs is another synthetic material, holding the network of organic ligands covalently bonded to each other. The advantages of COFs are somewhat similar to those of MOFs. Both MOFs and COFs are of permanent porosity and highly ordered structures. Their high degree of controllability in structure, synthesis and function make them the new favorites in the field of organic materials (Geng et al., 2020).

Researches about MOFs and COFs in *C. albicans* inhibitory applications are developing. Su et al. designed and synthesized voriconazole-Inbuilt zinc 2-methylimidazolates frameworks (V-ZIF) recently. Voriconazole is expected to be embedded in the MOF, and the former can only be responsive-released under the pH of 5 environment of the *C. albicans* biofilm. What’s more, they found that V-ZIFs provide better antifungal effect and antibiofilm effect than free voriconazole. This could be explained by V-ZIFs binding to *C. albicans* via electrostatic double-layer attraction, resulting in additional damage to the latter cell wall. The potential cytotoxicity of V-ZIPs and free drugs was compared using human embryonic kidney 293T (HEK 293T) cells and it was found that V-ZIPs yielded significantly less loss of cytotoxicity. V-ZIFs also could accelerate wound healing without obvious side effects in open wounds infected with *C. albicans* in infectious mice models (Su et al., 2020).

The applications of MOFs and COFs in drug delivery systems for antifungal purpose are still in its infancy, more progresses are expected in the future.

### Others

In addition to the antifungal effect of nanoparticles themselves and the use of nanomaterials as a carrier to improve the bioavailability of traditional antifungal drugs, some researchers have used nanomaterials to regulate macrophage-related immune processes to enhance the killing of *C. albicans* by the body itself. The conventional chemotherapeutic imatinib was encapsulated in dual functional mannosylated chitosan oligosaccharides which can decrease M2 macrophage population by inhibiting the STAT6 phosphorylation pathway and induce M1 macrophage polarization. This process will significantly increase the degree of resistance to *C. albicans* (Gao et al., 2020).

Photodynamic antimicrobial chemotherapy (PACT) is a new method fighting against bacteria and fungus, which mainly utilizes the absorbtion of specific wavelength of light activating photosensitizers to generate reactive oxygen species (ROS) such as singlet oxygen. Subsequently, ROS can induce a series of reactions and physical effects to achieve the purpose of killing microorganisms. PACT has been proved to be effective against *C. albicans* recently (Ikono et al., 2019). Khan et al. found that gold nanoparticles (AuNPs) could significantly promote the PACT mediated by methylene blue (MB), an classic photosensitizer, and increase the inhibitory rate of *C. albicans* biofilm from 81.9 to 95.4% (Khan et al., 2012).

## Conclusion

In recent years, *C. albicans* infection incidence and its drug resistance increased year by year, which make it become a global problem almost. Studies and literature on its infections and treatments are also emerging.

Nanomaterials have become a hot topic in the field of drug delivery systems due to their ability to carry, stabilize and protect therapeutic payloads and target cell binding. The antifungal effect of nanomaterials, such as inorganic nanoparticles, liposomes, polymeric nanoparticles, metal-organic frameworks (MOFs) and covalent organic frameworks (COFs) have been well verified in laboratory conditions. But there is still a long way to go before these materials can be put into clinical use. First of all, due to the high equipment and technology requirements and high cost of producing nanoparticles, it is difficult to achieve large-scale production at present, which limits its development in clinical application to some extent. Secondly, the specific mechanism, biological effects and toxic and side effects of nanoparticles are not clear. We still lack data on whether the nanoparticles themselves and their metabolites are truly environmentally and biologically benign. Moreover, Unlike other skin or digestive systems, the microenvironment of the female reproductive system changes periodically under the influence of reproductive hormones. Whether nanomaterials can adapt to such changes and give full play to their antifungal effects remains to be discussed. Many more laboratory and clinical trials are needed to resolve all these questions, but the promise of nanomaterials as antifungal treatments is unquestionable. In addition, currently, the nanocarriers themselves and their combinations with antifungal drugs or other nanomaterials are still limited. It may also be a promising research direction to find more and more effective combinations as possible.
